# The Hypoglycemic and Hypocholesterolemic Activity of *Dioscorea deltoidea*, *Tribulus terrestris* and *Panax japonicus* Cell Culture Biomass in Rats with High-Fat Diet-Induced Obesity

**DOI:** 10.3390/nu15030656

**Published:** 2023-01-28

**Authors:** Maria N. Povydysh, Maria V. Titova, Dmitry Yu. Ivkin, Marina V. Krasnova, Ekaterina R. Vasilevskaya, Liliya V. Fedulova, Igor M. Ivanov, Andrey G. Klushin, Elena V. Popova, Alexander M. Nosov

**Affiliations:** 1 Saint-Petersburg State Chemical Pharmaceutical University, Prof. Popov str. 14, Saint-Petersburg 197376, Russia; 2 K.A. Timiryazev Institute of Plant Physiology, Russian Academy of Sciences, Botanicheskaya str. 35, Moscow 127276, Russia; 3 V.M. Gorbatov Federal Research Center for Food Systems of Russian Academy of Sciences, Moscow 109316, Russia; 4 Faculty of Biology, Lomonosov Moscow State University, Leninskie Gory 1-12, Moscow 119991, Russia

**Keywords:** antiobesity, alimentary obesity model, bioimpedance spectroscopy, bioreactors, blood biochemistry, *Dioscorea deltoidea*, *Panax japonicus*, plant cell culture, phytopreparations, *Tribulus terrestris*

## Abstract

Obesity, and its consequences for human health, is a huge and complicated problem that has no simple solution. The constant search for natural and safe compounds with systemic action that can be used for obesity prophylactics and treatment is hampered by the limited availability and variable quality of biomass of wild medicinal plants. Plant cell biotechnology is an alternative approach for the sustainable production of vegetative biomass or individual phytochemicals with high therapeutic potential. In this study, the suspension cell biomass of the medicinal plants, *Dioscorea deltoidea* Wall., *Tribulus terrestris* L., and *Panax japonicus* (T. Nees) C.A. Mey, produced in 20 L and 630 L bioreactors, were tested for therapeutic effects in rat models with alimentary-induced obesity. Three-month intake of water infusions of dry cell biomass (100 mg/g body weight) against the background of a hypercaloric diet reduced weight gain and the proportion of fat mass in the obese animals. In addition, cell biomass preparation reduced the intracellular dehydration and balanced the amounts of intra- and extracellular fluids in the body as determined by bioimpedance spectroscopy. A significant decrease in the glucose and cholesterol levels in the blood was also observed as a result of cell biomass administration for all species. Hypocholesterolemic activity reduced in the line *P. japonicus* > *D. deltoidea* > *T. terrestris*/liraglutide > intact group > control group. By the sum of parameters tested, the cell culture of *D. deltoidea* was considered the most effective in mitigating diet-induced obesity, with positive effects sometimes exceeding those of the reference drug liraglutide. A safety assessment of *D. deltoidea* cell phytopreparation showed no toxic effect on the reproductive function of the animals and their offspring. These results support the potential application of the biotechnologically produced cell biomass of medicinal plant species as safe and effective natural remedies for the treatment of obesity and related complications, particularly for the long-term treatment and during pregnancy and lactation periods when conventional treatment is often contraindicated.

## 1. Introduction

The prevalence of obesity and the burden of obesity-related diseases are increasing worldwide [1,2]. According to WHO Global Health Observatory data, over 1.9 billion people in the world are overweight, from which nearly 650 million suffer from obesity with a body mass index (BMI) exceeding 30 kg/m^2^. Russia is amongst 10 countries with the highest obesity occurrence rate [3]. Various data sources estimate the ratio of obese and overweight adults in Russia from 20 to 50% [4], pointing also to the alarming increase in obese children and adolescents at the age of 5 to 19 [5,6]. Obesity is a major risk factor associated with the development of asthma, type 2 diabetes, site-specific cancers, cardiovascular diseases, musculoskeletal disorders, and complications after infectious diseases [7,8]. In particular, fat metabolism disorder is one of the factors increasing the disease severity and mortality risk in patients with SARS-CoV-2 (COVID-19). In hospitalized patients with COVID-19, the degree of obesity was also associated with the rate of progression of acute respiratory distress syndrome [9]. The increased BMI and obesity are linked to the development of reproductive disorders in both women and men, including an approximately 1.3 times higher risk of premature death, and may be a barrier to the use of assisted reproductive technologies [7,10,11,12,13].

Despite the critical role of obesity in human health, there is a growing number of contraindications and complications related to the treatment and the management of this disorder, including the use of medications. To date, only a few medications have received European Medicines Agency (EMA) approval for clinical use in the medical treatment of obesity, including orlistat, liraglutide, and the combination of bupropion and naltrexone. The availability of these medicines varies by country and may be subjected to restrictions under national regulations. In 2021, the U.S. Food and Drug Administration (FDA) also approved semaglutide for injection for chronic weight control in obese or overweight adults with at least one weight-related disease. However, this substance has not yet received the EMA approval [6,14].

Synthetic medications often have side effects and contraindications and are not always compatible with other medicines used for the long-term treatment of chronic disorders. For example, the administration of orlistat was related to negative gastrointestinal effects observed against a background of excess fat in the diet, several cases of severe liver damage, and decreased levels of fat-soluble vitamins [15,16]. Until recently, sibutramine was used in obesity treatment, but it has been withdrawn in the EU and USA due to a significant increase in cardiovascular outcomes such as nonfatal myocardial infarction and nonfatal stroke in obese or overweight patients with a history of cardiovascular disease during long-term sibutramine treatment [17]. Combined treatment with bupropion and naltrexone is contraindicated in the cases of uncontrolled arterial hypertension, epilepsy, anorexia, chronic use of opioid drugs, pregnancy, or in combinations with antidepressants [18]. Liraglutide is still considered one of the safest medications for the long-term treatment of obesity in combination with a low-calorie diet and increased physical activity, but it is restricted for patients with some forms of cancer, pregnancy, severe depression, severe liver and kidney function disorders, and acute pancreatitis [19,20]. A number of other medications for the treatment of obesity are currently under clinical trials [21]. Nevertheless, the choice of pharmacological strategy is still complicated due to the complex etiology of obesity and concomitant chronic diseases [22]. Therefore, reducing the side effects of the known medications and increasing the safety of therapeutic interventions, including long-term, for the most vulnerable patient groups remains an important task.

A promising approach for the treatment of obesity may include phytopreparations based on biologically active compounds from plant cells that demonstrate systemic effects on the human body [23,24,25]. The use of “phytomolecules” may be particularly relevant for patients with high contraindications for synthetic medications. Under optimum dosage and with known interactions with conventional pharmaceuticals, phytopreparations used in obesity treatments may reduce the duration of synthetic drug administration [26]. Compared to their synthetic counterparts, plant-derived compounds usually demonstrate a wider range of actions and fewer negative side effects [25,26,27]. Phytopreparations are rarely used as an emergency treatment for patients with severe conditions. However, the complex composition of herbal remedies implies a combination of effects, which make them effective during the treatment of concomitant and/or chronic diseases, especially for the long-term [28,29]. They may also be effective for the prevention of relapses, during recuperation, in preparation for pregnancy, and during the lactation period, which expands the therapeutic options [25,28].

The use of herbal preparations for the prevention and treatment of fat metabolism disorders and their complications has been reviewed [30,31]. Positive effects of plant extracts during the treatment of obesity involved different mechanisms such as suppressing appetite, reducing triglyceride levels, increasing the metabolic rate, inhibiting pancreatic lipase, stimulation of diuresis, regulation of lipid metabolism, stimulation of insulin secretion, action on the central nervous system through leptin, etc. [23,25,30]. However, significant variations in the quality of vegetative biomass remain a problem. The qualitative and quantitative content of pharmacologically important chemical components in plants may be species- and organ-specific and are subjected to change due to daily and seasonal fluctuations, environmental conditions, methods of cultivation, collection, processing and storage, etc. [32]. In addition, many medicinal plant species are rare or endangered, and their collection in the wild may lead to the depletion of their natural reserves. Biotechnological cultivation of plant cells and organs in vitro using large-scale bioreactor systems is a promising alternative approach to the production of ecologically safe and standardized plant biomass with a controlled and stable content of the desired phytomolecules [33,34]. This technology allows the design of herbal preparations that are unprofitable or impractical to produce using the traditional way of wild plant collection [34].

In the present study, we explored the therapeutic potential of the cell cultures of *Panax japonicus*, *Dioscorea deltoidea,* and *Tribulus terrestris,* medicinal plants that synthesize biologically active triterpene and steroid glycosides. Previous studies demonstrated that extracts of *Panax *spp**. plants containing ginsenosides affect the levels of leptin, ghrelin, and adiponectin, as well as help in reducing appetite and chronic hypothalamic inflammation, inhibit pancreatic lipase activity, which prevents the digestion and absorption of fats and carbohydrates, and reduce the glucose level in the blood. *Panax *spp**. may also have antiadipogenic effects and improve fat oxidation and energy expenditure [35,36,37,38,39,40]. *Tribulus terrestris* may be effective in treating hypercholesterolemia by reducing cholesterol, homocysteine, leptin, and resistin levels and increasing adipokine expression; it has also been shown to have hypolipidemic and antihyperglycemic activities [41,42,43,44]. *Dioscorea *spp.** were reported to reduce the total body weight (TBW) and weight of adipose tissue, and lower total cholesterol and triglyceride levels in the blood [45,46,47,48].

Biotechnological cultivation of the suspension cell cultures of *D. deltoidea*, *P. japonicus,* and *T. terrestris* in bioreactors of laboratory and industrial volumes has been previously developed [49,50,51]. However, the biological activities in cell cultures may differ from what was reported for their source plants due to potential alterations in secondary metabolite accumulation caused by the absence of organismic control and artificial growth conditions. Therefore, the bioreactor-derived cell biomass must be thoroughly investigated for its biological activities before it is certified for the use in any type of treatment [52,53].

In this study, we investigated the effects of phytopreparations based on the bioreactor-grown cell cultures of three medicinal plant species, *D. deltoidea, T. terrestris,* and *P. japonicus*, on laboratory rats with induced alimentary obesity. Phytopreparation from *D. deltoidea* cell biomass that demonstrated the most profound positive effect during the obesity treatment was further tested for the potential effects on the reproductive functions of laboratory rats.

## 2. Materials and Methods

### 2.1. Plant Suspension Cell Cultures, Bioreactor Cultivation, and Preparation of Cell Biomass

**Figure A1:**
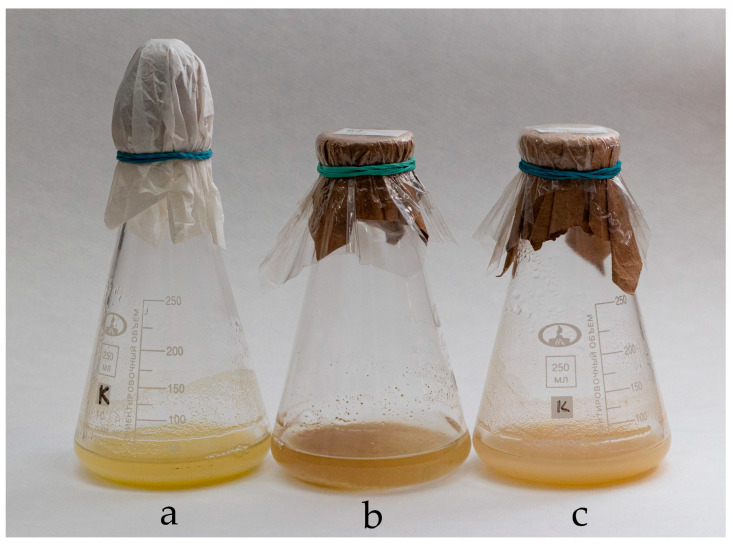
Stock cultures of (**a**) *Dioscorea deltoidea*, strain DM-05-03, (**b**) *Panax japonicus*, strain 62, and (**c**) *Tribulus terrestris*, strain Tter8 maintained in the All-Russian Collection of Plant Cell Cultures (Institute of Plant Physiology of Russian Academy of Sciences, Moscow).

**Figure A2:**
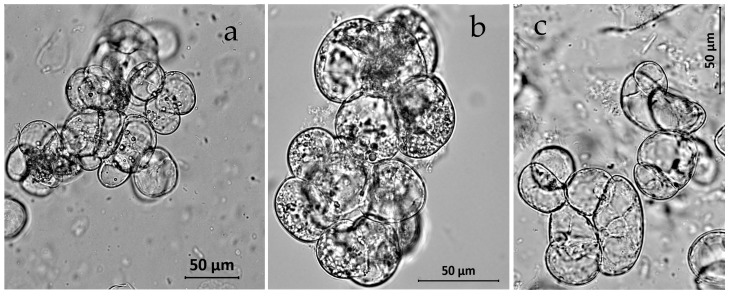
Microscopic photographs of the suspension cultures of (**a**) *Dioscorea deltoidea*, strain DM-05–03, (**b**) *Panax japonicus*, strain 62, and (**c**) *Tribulus terrestris*, strain Tter8.

1. Suspension cell cultures of medicinal plants with enhanced production of active metabolites were received from All-Russian collection of plant cell cultures, Institute of Plant Physiology of Russian Academy of Sciences (Appendix A, Figure A1 and Figure A2). The following cell cultures were used in the study:
2. Suspension cell culture of *Dioscorea deltoidea* Wall. ex Griseb., strain DM-05-03, total content of furostanol glycosides (25 (S)-protodioscin, protodioscin and deltoside) 4.62% of dry cell weight (DW);
3. Suspension cell culture of *Panax japonicus* (T. Nees) C.A. Mey., strain 62, total content of ginsenosides (Rg1, malonyl-Rg1, Rb1, malonyl-Rb1, Rb2/Rb3, malonyl-Rb2/Rb3, Rd, malonyl-Rd, Rf, R0, chikusetsusaponin IVa) 3.46% DW;
4. Suspension cell culture of *Tribulus terrestris* L., strain Tter8, total content of furostanol glycosides 0.1% DW.

The analysis of content and composition of bioactive secondary metabolites in the cell cultures was described previously [50].

**Figure A3:**
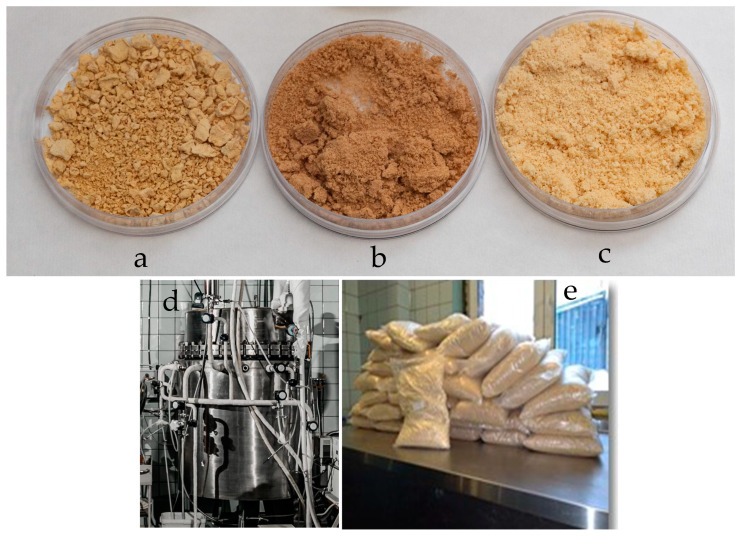
(**a**–**c**) Bioreactor-grown and air-dried biomass powder of (**a**) *Dioscorea deltoidea*, strain DM-05-03, (**b**) *Panax japonicus*, strain 62, and (**c**) *Tribulus terrestris*, strain Tter8; (**d**) 630-l bioreactor for cultivation of plant cell suspensions; (**e**) dried cell biomass from a 630-l bioreactor.

Cell suspensions were cultured in 20 L (*T. terrestris*) and 630 L (*D. deltoidea* and *P. japonicus*) bubble-type bioreactors under semi-continuous regime at 25 ± 1 °C in darkness (Appendix A, Figure A3). Cultivation conditions for each culture including medium composition, agitation, oxygen concentration, inoculum density etc. were described earlier [49,54,55]. Cell biomass was harvested from bioreactors at the point of maximum dry weight accumulation and separated from liquid medium on Nutsche filters under vacuum, then rinsed three times with distilled water. The resulting cell mass was dried on stainless-steel shelves at 45 ± 1 °C for 18 h. This dried cell powder (Appendix A, Figure A3) was used to prepare aqueous suspensions by infusing dried biomass into purified water at a ratio of 1:20 at room temperature for 7 h [50]. These suspensions were orally administrated to laboratory animals following the experimental scheme as described below.

### 2.2. Anti-Obesity Effects of Cell Biomass Preparations of D. deltoidea, T. terrestris, and P. japonicus

#### 2.2.1. Laboratory Animal Husbandry

Inbred male Brown Norway rats were used in this study. The animals were kept under standard conditions in accordance with «OECD. Principles of good laboratory practice». The animals were provided with standard feed (“Complete feed for laboratory animals”, LLC “Laboratorkorm”, Moscow, Russia) and water (GOST 2874–82 “Drinking water”) *ad libitum*. Access to food was restricted prior to blood collection. Before the study, animals were quarantined in a separated room for 14 days. Each animal was assigned an individual number (a mark on the tail area with a permanent indelible marker, periodically updated).

#### 2.2.2. Experimental Model of Alimentary Obesity and Antiobesity Effects of Cell Biomass Preparations

The animals were randomly divided into two groups: group 1 (*n* = 10) was an intact group receiving standard feed (SF); group 2 (*n* = 50) served to model alimentary obesity and received hypercaloric feed (HF) with an excess of easily digestible carbohydrates (Table 1). Hypercaloric feed was composed of standard feed (63%) with addition of rendered beef fat (19%), sucrose (10%), and isolated soy protein (8%) [56].

After three months on HF diet, changes in the body parameters of the animals including body weight (BW), fat mass (FM), fat-free mass (FFM), total body water (TBW), the amount of intra- and extracellular fluid (ICF and ECF), and BMI were analyzed by bioimpedance method (see below) [57,58]. Formation of obesity in group 2 rats receiving HF was confirmed by a significant increase in fat mass and BMI compared to group 1 receiving SF (Table 2).

During the next 3 months, subgroup 1 (intact animals) continued to receive standard feed while animals of group 2 with developed obesity were divided into subgroups 2 to 6 (Table 1). Subgroup 2 continued to receive HF only and served as an untreated control. Subgroup 3 received HF and daily subcutaneous injections of a reference drug liraglutide (Saxenda ©, Novo Nordisk, Denmark) (0.3 mg/kg in 1 mL 0.9% NaCl). Subgroups 4–6 received HF and phytopreparations (Table 1). Phytopreparations were administered intragastrically in concentrations equivalent to 100 mg dry cell biomass per kg animal weight once a day. The doses of liraglutide and phytopreparations used in this study were selected based on previously published recommendations [50,59,60].

The effects of the phytopreparations and liraglutide on the obese rats were evaluated after 6 months (including 3 months of obesity formation followed by 3 months of treatment with liraglutide or phytopreparations) using several parameters measured by bioimpedance spectroscopy (BIS) including fat mass, fat-free mass, total body water, the amount of intra- and extracellular fluid, as well as body weight, blood glucose, total cholesterol, and daily urine output (see below).

#### 2.2.3. Bioimpedance Spectroscopy (BIS), Collection, and Evaluation of Biological Material

**Figure A4:**
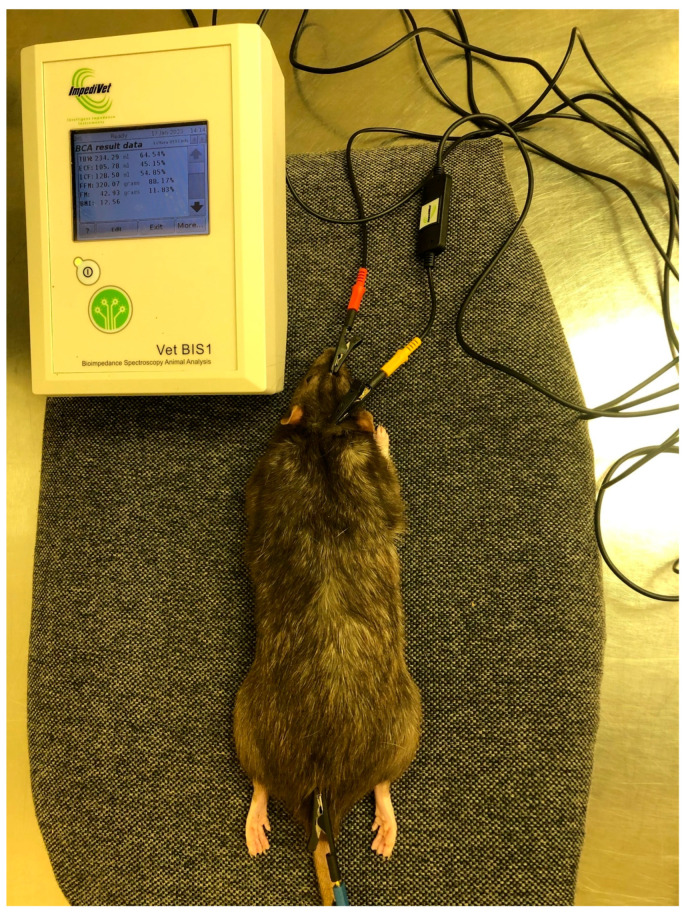
Bioimpedance spectroscopy (BIS) analysis.

Bioimpedance spectroscopy, a noninvasive method for the estimation of body composition [57], was performed using ImpediVet BIS1 spectroscopic system (ImpediMed, USA). The device allows accurate measurement of the total body weight, extracellular and intracellular fluid volumes, fat-free mass, and fat mass, as well as body mass index. ImpediVet is a single-channel tetrapolar device that scans 256 frequencies from 4 to 1000 kHz. Low-frequency current passes through extracellular fluids but not through cell membranes; hence, the impedance at low frequencies relates to ECF volume. High-frequency current can travel through cell membranes and passes through both extracellular fluids and cells, thus relating to TBW. Calculation of ICF was performed by subtracting ECF from TBW [57]. Evaluation of fat mass and lean body mass is based on the difference in the water composition of muscles and adipose tissues [58]. The animals were anesthetized by a mixture of zoletil (25 mg/kg) and xylazine (10 mg/kg) and placed on a heated platform to maintain body temperature. Electrodes equipped with needles were inserted in the area of the nose, between the ears, at the base of the tail, and the coccygeal zone (Appendix A, Figure A4). Three consecutive measurements were performed on each animal with an interval of 3 s. The following constant parameters associated with the type of laboratory animals were used by default: body proportion (length to girth ratio) 1.0; body density 1.05 g/cm^3^; hydration constant 0.732; resistance coefficients ρ_i_ = 325; ρ_e_ = 752 [59].

Blood sampling was performed in alive animals by incising the gum between the lower incisors. After fixing the rat by hand with a sharp blade, a small incision was made in the gums (mixed blood). The blood was applied to glucometer test strips and/or collected in vacuum tubes with a blood coagulation activator. Blood glucose concentration was measured using Accu-Chek Active glucometer (Roche Diagnostics, Switzerland) with an accuracy of 0.1 mmol/L.

To measure total cholesterol (TC) level, blood sampling was performed in alive animals after an overnight (10–12 h) fast. After taking blood from the gums into Vacuette vacuum tubes with a blood coagulation activator, the tubes were left for 30 min to settle, then the blood was centrifuged for 10 min at 1000 rpm, the resulting serum was separated, and then centrifuged again for 15 min at 4000 rpm. The analysis of the serum was performed using a biochemical blood analyzer (Erba xl100 (Erba Lachema, Czech Republic).

Urine was collected within 24 h in metabolic cages ensuring the separation of feces and urine. The volume of daily urine was determined using laboratory volumetric glassware.

### 2.3. Effect of D. deltoidea Cell Biomass Preparation on Reproductive Functions of Laboratory Rats

From three cell lines tested for obesity treatments described in Section 2.2, phytopreparation from *D. deltoidea* cell biomass demonstrated the most profound positive effect. This phytopreparation was further tested for its potential effects on reproductive functions of laboratory rats.

Experiments were performed on clinically healthy SPF Wistar rats. Rats of the initial colony (generation F0), males and females, weighing 220 ± 15 g were randomly selected from the stock maintained at Animal Breeding Facility of BIBCh, RAS (the Unique Research Unit Bio-Model of the IBCh, RAS; the Bioresource Collection-Collection of SPF-Laboratory Rodents for Fundamental, Biomedical and Pharmacological Studies), which has an AAALACi accreditation. Animal husbandry conditions during the whole experiment were described earlier [49].

After 14 days of adaptation, F0 rats were divided into 2 groups (*n* = 14), experimental and control. Rats of the experimental group received a phytopreparation of *D. deltoidea* cell biomass which was supplied daily at the dose of 100 mg biomass/kg through polysulfone drinkers (Techniplast, Italy) for 80 days.

Reproductive function was evaluated by assessing the fertility of F0 animals and the postnatal development of F1 generation. For fertilization, female rats (*n* = 7 in each group) were housed together with males in a ratio of 2:1 for 14 days. The pregnancy of one or both females confirmed the fertility of the male. Fertility was determined as a percentage of the females that became pregnant and the males with fertilization ability out of total number of females/males. The average litter size, male-to-female ratio in the litter, and the number of live and dead newborns were also determined. Pup survival rate was determined twice: (i) from day 0 to day 5 as the percentage of pups survived by day 5 out of live newborns, and (ii) from day 6 to day 25 as the percentage of pups survived by day 25 out of number of pups alive at day 6.

The postnatal development of F1 generation was evaluated during the first 30 days of life by the number of live and dead newborns, dynamics of body weight, and general physical development [61,62,63]. Rat body weight was measured using Adventurer Pro electronic technical scales (Ohaus, Parsippany, NJ, USA) with an accuracy of ±0.1 g on days 4, 7, 11, 14, 18, 22, and 25 after birth.

At the end of the experiment (80 days), females of parental generation (F0) and generation F1 (30-day-old) were euthanized in the carbon dioxide chamber (VetTech, Congleton, UK), blood was collected, and general autopsy was performed.

Quantification of erythrocytes and platelets in the blood was performed on a fully automatic hematology analyzer Abacus junior vet 2.7 (Diatron Messtechnik GmbH, Austria) using Diatron reagent kits. Lymphocyte (LYM), granulocyte (GRA), and monocyte (MON) contents were determined on a Guava Easy Cyte flow cytometer (Merck Millipore, Germany) by detecting cell size and granularity. Total leukocyte count (WBC) was determined using the formula: WBC = LYM + GRA + MON. The relative content of lymphocytes, granulocytes, and monocytes was determined according to the formulas: LYM/WBC*100%, GRA/WBC*100%, MON/WBC*100%.

Biochemical parameters of blood serum were analyzed on a BioChem FC-360 automatic biochemical analyzer (HTI, Farmington, MI, USA) using HighTechnology reagent kits. The antioxidant status of the blood serum was evaluated as described earlier [64]. The level of malondialdehyde (MDA), the major lipid peroxidation product, in plasma was evaluated using the thiobarbituric acid reactive substances (TBARS) assay. Catalase (CAT) activity in blood plasma was determined on a spectrophotometer SF-2000 (OCB «Spectr», Russia) according to [65]: 720 µL of 50 mM phosphate buffer (pH = 7.0) was mixed with 800 µL of 0.1% hydrogen peroxide and absorbance (D0) was measured (wavelength 240 nm, 1 cm cuvettes) relative to the control sample. Next, 20 µL of plasma were added to the tubes and the absorbance (D1) was measured after 1.5 min of incubation. In the control, 800 µL of phosphate buffer was added instead of hydrogen peroxide. Concentration of reduced glutathione and glutathione reductase activity in serum was determined on a spectrophotometer SF-2000 (OCB «Spectr», Russia) using the Ellman’s reagent: 5,5′-dithiobis-(2-nitrobenzoic acid) (DTNB) dissolved in methanol. Total antioxidant activity was determined by the rate of the oxidation of the reduced form of 2,6-dichlorophenolindophenol (DCPIP) in the reaction medium using a spectrophotometer (HTI, HTI, Farmington, MI, USA) [65]. The de Ritis coefficient was calculated as the ratio of aspartate aminotransferase (AST) and alanine aminotransferase (ALT) activity according to the following formula [66]:

(1) $$\mathrm{de}\;\mathrm{Ritis}\;\mathrm{coefficient}=\frac{\mathrm{AST}}{\mathrm{ALT}}$$

Autopsy of animals of all groups included a thorough examination of the external surface of the body, all orifices, intracranial, thoracic, and abdominal cavities, and their contents.

The research was approved by the bioethical commission of the V.M. Gorbatov Federal Research Centre for Food Systems of the Russian Academy of Sciences.

### 2.4. Statistical Data Analysis

Statistical analysis of the data obtained in the experiments on antiobesity effect of phytopreparations was performed using the GraphPad Prism 8 software package (GraphPad Software, San Diego, CA, USA). When assessing the significance of differences between the study groups, the hypothesis of the normal distribution of the trait was tested using the Kolmagorov–Smirnov test. In the case of a normal distribution, the parametric Student’s t-test (*p* < 0.1) was used, otherwise the nonparametric Mann–Whitney tests [67] were applied at *p* < 0.1. Data in figures and tables are presented as means followed by standard deviations.

Statistical analysis of data on phytopreparation effect on fertility was performed using STATISTICA 10 software. Results were presented as mean ± standard deviation, minimum and maximum values, or percentiles (P25–P75) depending on the dataset. Statistical validity was calculated using one-way ANOVA with Tukey’s test at *p* < 0.05.

## 3. Results

### 3.1. Antiobesity Effects of Cell Biomass Preparations

#### 3.1.1. Alimentary Induced Obesity Model

As mentioned above, a hypercaloric, high-fat diet with an excess of digestible carbohydrates was used to model alimentary obesity. The rats receiving HF weighed 23.64% more than the animals on the standard diet (Table 2). After three months of receiving a hypercaloric diet, the fat mass of the animals and BMI were significantly higher, and the fat-free mass was significantly lower than in the group receiving the standard feed (Table 2). The total amount of water in the body decreased in the animals fed with hypercaloric food (Table 2). Simultaneously, we observed a decrease in ICF and an increase in ECF in rats on HF, which reflected a tendency to the intracellular dehydration.

After six months of the experiment, changes in animal body weight and blood glucose level were analyzed, and the results for subgroups 1–3 are given in Table 3 and Table 4. The final weight change was 12.57% in group №2 and −22.41% in group №3 compared to the intact animals of subgroup №1 (Table 3). The data presented in Table 2, Table 3 and Table 4 confirm that alimentary-induced obesity was successfully reproduced in the rat model according to the selected criteria [67]. Liraglutide effectively reduced both weight gain (Table 3) and the blood glucose level (Table 4) which justified its choice as a reference drug.

#### 3.1.2. Hypoglycemic and Hypocholesterolemic Activity in Liraglutide and Phytopreparations of *D. deltoidea*, *P. japonicus*, and *T. terrestris* Cell Cultures

**Figure 1:**
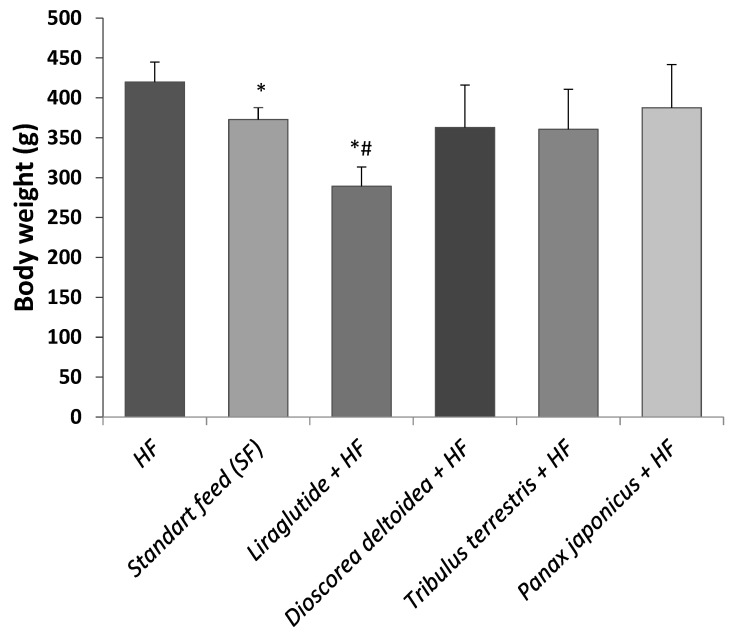
Body weight of the animals of intact (SF), control (HF), and experimental groups measured at the end of the experiment (3 months of obesity formation followed by 3 months of treatments). SF—standard feed, intact group; HF—hypercaloric feed. *—significantly different from the control (HF) group; #—significantly different from the intact (SF) group (*, #–0.01 < *p* < 0.05).

In the induced obesity model, treatment with phytopreparations effectively reduced the accumulation of body weight (Figure 1) compared to animals on HF without any treatment. However, their action was milder compared to the reference drug liraglutide.

**Figure 2:**
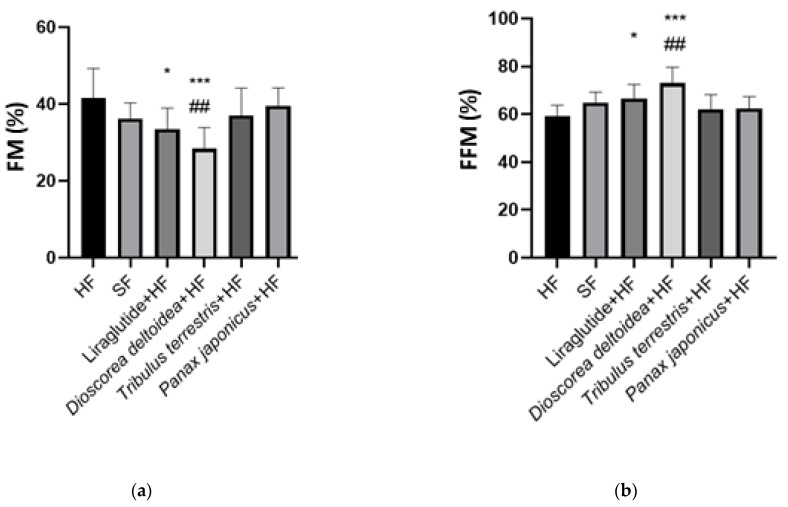
Fat mass (FM, (**a**)) and fat-free mass (FFM, (**b**)) values of the animals of intact, control, and experimental groups measured at the end of the experiment (3 months of obesity formation followed by 3 months of treatments). SF—standard feed, intact group; HF—hypercaloric feed. *—significantly different from the control (HF) group; #—significantly different from the intact (SF) group (*—0.01 < *p* < 0.05; ##—0.001 < *p* < 0.01; ***—*p* < 0.001).

The three-month treatment of obese animals with the cell biomass of *D. deltoidea* resulted in a significant decrease in the proportion of body fat mass (28.37 ± 5.49%) compared to both the control (41.5 ± 7.72%, *p* = 0.0007) and intact (36.23 ± 4.1%, *p* = 0.0034) groups (Figure 2). Consequently, there was a significant increase in FFM in the group of obese animals that received *D. deltoidea* cell biomass against the background of a hypercaloric diet (73.05 ± 6.70%) relative to the control (59.20 ± 4.61%, *p* = 0.0001) and intact (64.95 ± 4.07%, *p* = 0.0001) animals (Figure 2). Interestingly, these parameters were even better in the cell biomass-treated animals than in the animals of the intact group.

**Figure 3:**
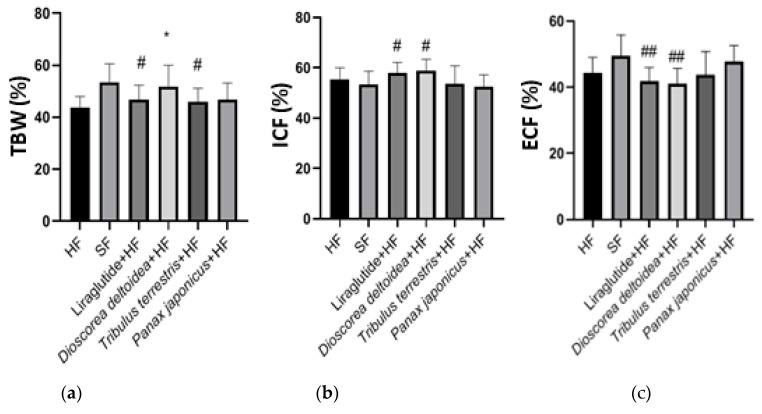
Total body water (TBW, (**a**)), intracellular fluid (ICF, (**b**)), and extracellular fluid (ECF, (**c**)) values in animals of intact, control, and experimental groups measured at the end of the experiment (3 months of obesity formation followed by 3 months of treatments). SF—standard feed, intact group; HF—hypercaloric feed. *—significantly different from the control (HF) group; #—significantly different from the intact (SF) group (*, #—0.01 < *p* < 0.05; ##—0.001 < *p* < 0.01).

Rats that were injected with liraglutide against the background of a hypercaloric diet also demonstrated a significant decrease in FM and an increase in FFM when compared with the control HF group: 36.07 ± 7.60% vs. 41.50 ± 7.72% (*p* = 0.0209) for FM and 71.62% ± 10.13% vs. 63.93% ± 7.60% (*p* = 0.0462) for FFM (Figure 3). These values were close to the animals of the intact group.

The FM and FFM of the animals treated with the cell biomass of *P. japonicus* and *T. terrestris* laid in between the control (HF) and intact (SF) groups, i.e., these preparations caused a slight decrease in the fat mass accumulation of the obese animals, but the differences from the control group without treatment were insignificant (Figure 2).

The hypercaloric feed led to a notable decrease in the percentage of the total body water and ECF in rats compared to the group with the standard feed (Figure 3). Animals that received liraglutide treatment against the background of a hypercaloric diet also showed a significantly lower TBW and ECF compared to the intact group: 46.80 ± 5.57% vs. 52.43 ± 7.42% (*p* = 0.0372) for TBW and 41.89 ± 4.07% vs. 49.54 ± 6.30% (*p* = 0.0047) for ECF, but the differences from the control (HF) group were insignificant. The amount of the intracellular fluid of the animals receiving liraglutide was slightly higher than in the intact group: 58.11 ± 4.07% vs. 53.49 ± 5.13% (*p* = 0.0387).

The total body water content in the animals receiving the cell biomass of *D. deltoidea* was significantly higher compared with the control group: 51.92% ± 8.08% vs. 43.76% ± 4.18% (*p* = 0.0161) and similar to the intact group (Figure 3). In addition, both the ICF and ECF values in the *Dioscorea*-treated rats were slightly different from the intact group, and the balance was shifted toward intracellular fluid accumulation. Indeed, the amount of intracellular fluid in the *Dioscorea* animal group exceeded the amount of extracellular fluid: 58.87% ± 4.58% vs. 41.13% ± 4.32% (Figure 3). Administration of the *T. terrestris* and *P. japonicus* cell biomass did not cause any significant changes in the TBW and fluid distribution compared to the control HF group (Figure 3).

**Figure 4:**
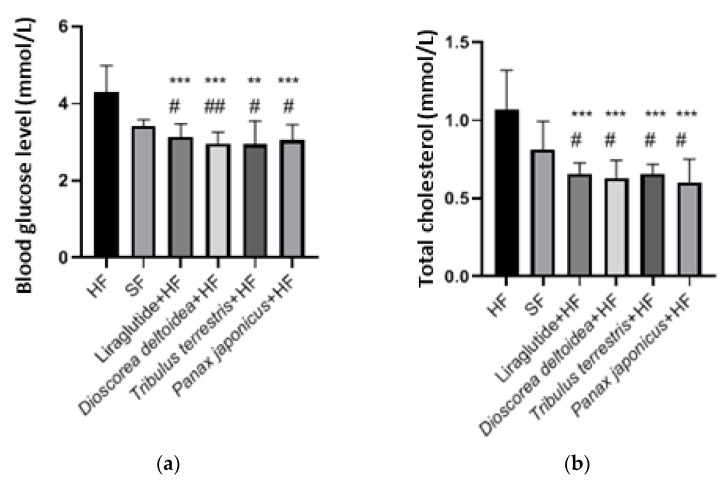
Blood glucose level (**a**) and total cholesterol in blood serum (**b**) in animals of intact, control, and experimental groups measured at the end of the experiment (3 months of obesity formation followed by 3 months of treatments). SF—standard feed, intact group; HF—hypercaloric feed. *—significantly different from the control (HF) group; #—significantly different from the intact (SF) group (#—0.01 < *p* < 0.05; **, ##—0.001 < *p* < 0.01; ***—*p* < 0.001).

In this study, phytopreparations and liraglutide showed hypocholesterolemic and hypoglycemic effects (Figure 4). The most pronounced effect on the glucose level in the blood serum was noted in the animal groups that received the cell biomass of *D. deltoidea* (2.96 ± 0.29 mmol/L) and *T. terrestris* (2.92 ± 0.65 mmol/L) (Figure 4). The highest cholesterol-lowering activity was recorded for the cell biomass of *P. japonicus* (0.60 ± 0.015 mmol/L) in comparison with the control (*p* = 0.0004) and intact (*p* = 0.0240) groups (Figure 4).

**Figure 5:**
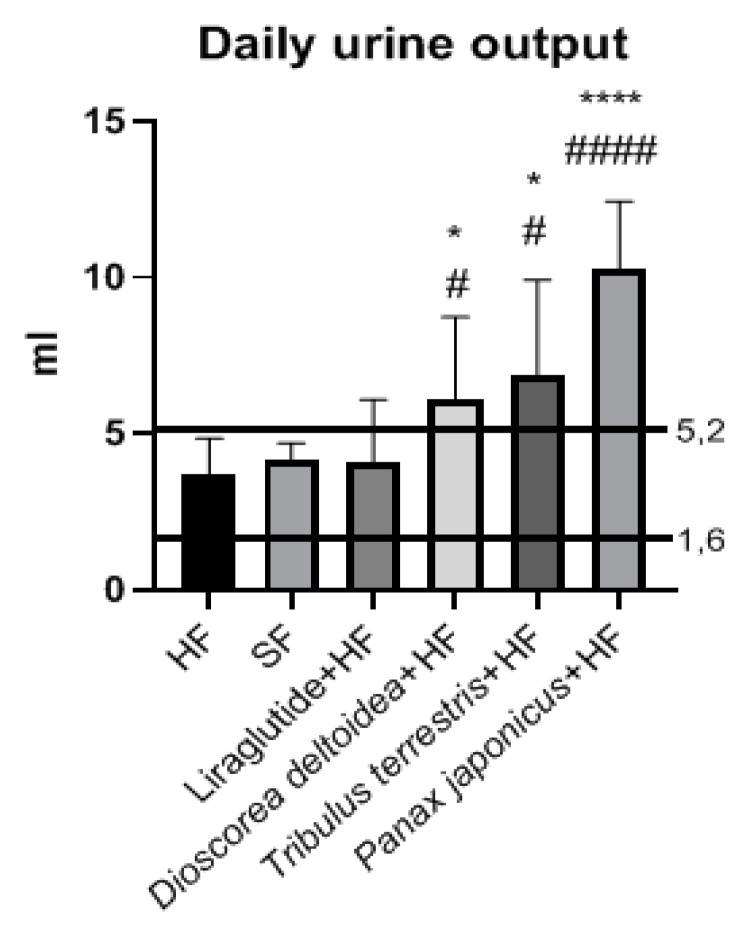
Daily urine output in animals of intact, control, and experimental groups. SF—standard feed, intact group; HF—hypercaloric feed. *—significantly different from the control (HF) group; #—significantly different from the intact (SF) group (*, #—0.01< *p* < 0.05; ****, ####—*p* < 0.001).

The daily urine output in the intact and HF groups recorded before drug administration (3 months after the beginning of different feeding) ranged from 1.6 mL to 5.2 mL (indicated in Figure 5 by two horizontal lines). At the end of the experiment (after 3 months of treatment), an increase in the daily urine output was observed in the animals that received the cell biomass of *D. deltoidea, T. terrestris,* and *P. japonicus* compared to the control, intact, and liraglutide-receiving groups (Figure 5). The daily urine output of the rats treated with the *P. japonicus* cell biomass was more than two times higher than in the intact group and added up to 10.3 ± 2.2 mL. In the groups treated with the *D. deltoidea* and *T. terrestris* cell biomass, the daily urine volume was nearly similar (6.8 ± 3.1 mL and 6.1 ± 2.7 mL, respectively). In the rats that were injected with liraglutide against the background of a hypercaloric diet, no statistically significant change in the daily urine output (4.1 ± 2.0 mL) in comparison with the control and intact groups was observed (Figure 5).

In conclusion, phytopreparation from the *Dioscorea deltoidea* cells was the most efficient in normalizing the fat mass and liquid balance in animals with induced obesity. All three types of phytopreparations tested were comparable with the reference drug liraglutide in reducing the glucose and cholesterol level in the blood. Administration of cell biomass also increased urine output. Based on these results, the phytopreparation of *D. deltoidea* was selected for further analysis of its potential effect on the reproductive functions and antioxidant activity in rats.

### 3.2. The Analysis of Possible Toxic Effects of D. deltoidea Phytopreparation on Reproductive Functions

The fertility of both females and males in the control and experimental groups was 100%. No birth-related mortality was observed in the F0 females of both groups.

A comparative analysis of the F0 rats mating results is presented in Table 5. The total number of newborn pups in the experimental group was 12% lower compared to the control group. The experimental group showed a lower variation in the average litter size (7–9 pups) compared to the control group (4–11 pups). The ratio of males and females in the experimental group was nearly equal while in the control group the number of males was 1.7 times higher than females. In both groups, the variations in reflex development were typical for the rats of this line (Table 5).

**Figure 6:**
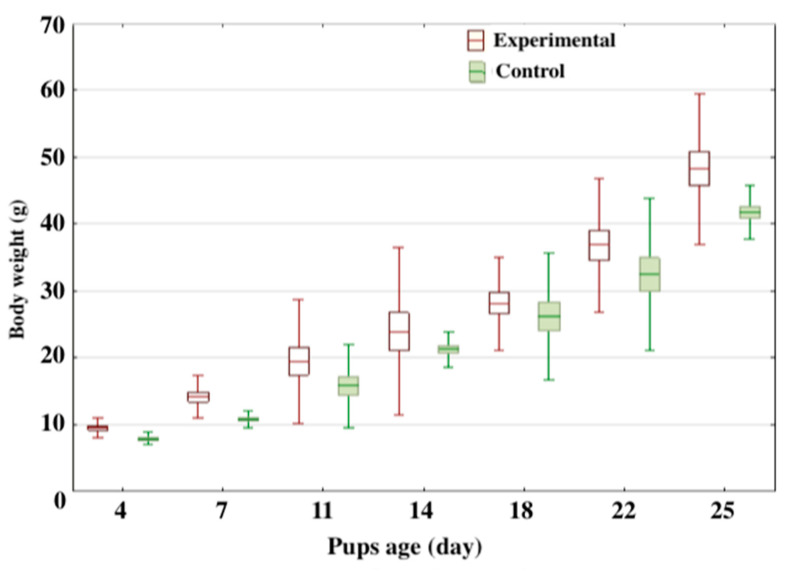
The dynamics of the rat pups’ body weight accumulation. Control—intact parents; Experimental—parents received the phytopreparation of *Dioscorea deltoidea* cells. Data are presented as box plots where the box illustrates the value range from the 25th to 75th percentiles with the median line in the middle, and the bars extend to the minimum and maximum values of the dataset.

Analysis of the weight dynamics in the F1 generation (Figure 6) revealed that the pups of the experimental group whose parents received the phytopreparations of *D. deltoidea* gained weight more intensively than the animals of the control group during the whole period of observation. This tendency was the most pronounced from the 4th to 7th day and by the 25th day. The increase in body weight during this period in the animals of the experimental group reached 49% in comparison with 36% in the control group.

Analysis of the hematological test panel revealed no significant changes between the control and experimental groups of the F0 (parent) and F1 rats. The only significant increase was observed in the leukocyte content in the F0 rats, mostly due to lymphocytes (up to 30%), while the content of immature leukocyte forms, granulocytes and monocytes, remained at the control level (Table A1). Among the indices of the functional activity of erythrocytes (Table A2), a slight decrease was recorded in hematocrit while the concentration of hemoglobin in erythrocytes increased (up to 4%). No statistically significant changes between the groups were found with regard to the platelet component (Table A2).

The comparative analysis of the blood serum biochemistry (Table A3) revealed that the administration of *D. deltoidea* caused a significant increase in alanine aminotransferase activity (by over 15%), gamma-glutamyltranspeptidase (up to 40%), and urea (by 10%) in the F0 parent rats compared to the control. Minor increases in the activity of aspartate aminotransferase, alkaline phosphatase, and lactate dehydrogenase (up to 10%) were statistically insignificant. At the same time, the de Ritis ratio also did not significantly differ between the groups. In the experimental F1 rats, a nonsignificant increase in the total protein content (by 7%, mostly due to the albumin fraction) and a significant increase in creatinine (by 11%) were recorded compared to the F1 control group (Table A3). Minor insignificant decreases in aspartate aminotransferase and alkaline phosphatase activity (to 15% and 7%, respectively) were also detected.

The analysis of the antioxidant potential of the blood (Table 6) demonstrated a significant decrease in the total antioxidant activity of the blood (by 7%) in the rats of the F1 experimental group alongside an elevated malondialdehyde content (over 10%), reduced glutathione (up to 30%), and catalase activity (up to 40%). In the F1 generation, a significant increase in the total blood antioxidant activity (by 15%) and reduced glutathione (up to 40%) were detected in the experimental group compared to the control.

It should be noted that the observed fluctuations in the hematological and biochemical blood parameters were within the physiological norm for this animal type.

Based on the results of the autopsy, macroscopic examination, and analysis of the relative weight of the internal organs, no differences were found between the animals of the experimental and control groups of the F0 and F1 generations (Table A4).

## 4. Discussion

Bioreactor-grown plant cell cultures are frequently acknowledged as a sustainable and renewable source of vegetative biomass rich in valuable phytochemicals. However, the phytochemical composition of such biomass may be altered compared to their respective donor plants. This, for example, was the case in the *Digitalis lanata* cell culture which contained a complex mixture of phenylethanoid compounds, including digiciliside A, digiciliside B, maxoside, purpureaside E, and their methyl derivatives and isomers, as well as seven furostanol glycosides with aglycones tigogenin and gitogenin but not cardiac glycosides that are major bioactive metabolites of the plants [68]. Phytoecdysteroid composition in the cell cultures of *Ajuga turkestanica* was narrowed compared to hairy roots or whole plants while a number of phenylpropanoids were detected in callus cultures [69,70]. Significant differences in the composition of phenolic compounds were revealed between the in vitro cell cultures and plant roots of *Phlojodicarpus sibiricus*, an endangered endemic species of Eastern Siberia [71]. The ratio of furostanol and spirostanol-type glycosides differed between the plant rhizomes and several cell culture strains of *Dioscorea deltoidea* [72]. The cell culture of *Panax japonicus* used in the present study accumulated high levels of “acidic” ginsenosides (malonyl derivatives of dammarane ginsenosides and glycosides of oleanolic acid) in contrast to the plant roots where neutral ginsenosides are usually prevailing [73]. Phenylethanoids are major compounds in the cell culture of *Tribulus terrestris*, but only trace amounts of these chemicals are usually found in plants [74]. Therefore, there is a nonzero chance that cell biomass will show different biological actions compared to what was reported for plants of the same species. In view of these differences, the biological activity cell-culture-based phytopreparations or their crude extracts should be thoroughly investigated before they could be recommended for use.

### 4.1. Antiobesity Effects of Cell Biomass Preparations of D. deltoidea, T. terrestris, and P. japonicus

A hypercaloric, high-fat diet with an excess of easily digestible carbohydrates was used to model alimentary obesity. This diet affects fat and carbohydrate metabolism in animals, in particular contributing to the development of liver steatosis.

There are many experimental models of obesity: genetic, neuroendocrine, surgical, diet-induced, and others. However, the diet-induced obesity is believed to most closely resemble the obesity in humans based on both etiology and developmental mechanisms. Among diet-induced obesity models, high-fat, high-carbohydrate and combined high-fat and high-carbohydrate models are the most commonly used [75,76]. High-fat and high-carbohydrate models are not very effective, and obesity develops slowly on high-carbohydrate/low-fat models [77]. Combined diets (“cafeteria diet”, “western diet”) do not always allow clear differentiation between the obesity-induced disorders and disorders caused by the deficiency of vitamins and minerals [78,79]. Therefore, in this study, a hypercaloric, high-fat diet was used to induce obesity [78,80]. Animal fats used in such diets are rich in saturated fatty acids that usually cause a more rapid and profound weight gain than the same amounts of polyunsaturated fats [77,81]. Male rats of the wild type are often used in the obesity studies [82,83] since they are omnivores like humans, have similar taste buds, food identification, and digestion systems. Neuroanatomically, they are also close to humans in terms of the brain areas that control food intake [84]. According to several researchers, diet-induced models on the wild rat type are closer to the pathogenesis of human obesity than models on genetically modified animals which can develop obesity on conventional diets [84,85].

In our study, the bioimpedance analysis clearly demonstrated the developed differences in the body composition between rats receiving hypercaloric or standard feed. The BIS method is relatively noninvasive and shows excellent reproducibility when assessing the body condition in dynamics, which is particularly important while modeling alimentary obesity in vivo [86]. There was a direct significant relationship between the excess body weight and fat mass. According to the results of our study, there was an increase in the fat mass and, consequently, in the body weight of the animals receiving a hypercaloric diet. The weight gain was mostly due to an increase in the fat mass and less due to an increase in the muscle mass. As obesity progressed, the animals receiving the hypercaloric feed had a decreased total body water (by ca. 20%) compared to the animals on standard feed, mostly due to intracellular dehydration.

Liraglutide was chosen as a reference drug due to the absence of the similarly effective herbal remedies for obesity treatment. Liraglutide also has an advantage over orlistat and sibutramine due to its pleiotropic effects, including hypoglycemic action, proven safety profile, and absence of excitatory activity. Liraglutide administered at 200 mg/kg twice a day significantly reduced the blood glucose, serum total cholesterol, TG, and low-density lipoprotein (LDL) cholesterol. It also reduced the intensity of lipid deposition in the liver in the db/db mice receiving a hypercaloric diet [87]. Bugáňová et al. [88] reported that liraglutide treatment reduced glucose levels by 1.8 times and triglycerides by 28% against a background of fasting compared to the control group of mice. Similarly, in the present study, liraglutide reduced the cholesterol content in the blood serum of the obese animals by 36% and glucose levels by 42% compared to the animals without treatment. Hansen et al. [89] found that liraglutide significantly reduced the body weight of the male Sprague Dawley rats with developed alimentary-induced obesity. Similar to our study, the drug promoted a decrease in the fat mass in rats on a hypercaloric diet [89]. In the study of Bugáňová et al. [88], liraglutide demonstrated the ability to reduce the body weight of animals with HCD-induced obesity. In our study, liraglutide contributed to a 19.5% reduction in the fat mass in Brown Norway rats with induced obesity.

As discussed earlier, the effectiveness of plant cell biomass extracts against induced obesity was assessed using bioreactor-produced cell cultures of the medicinal plants *Panax japonicus*, *Dioscorea deltoidea,* and *Tribulus terrestris* with bioactive secondary metabolites: triterpene and steroid glycosides. Plant extracts of these species show a wide range of biological actions, including reduction in body weight and appetite suppression, effects on leptin, ghrelin, and adiponectin levels, inhibition of pancreatic lipase activity which prevents digestion and absorption of fats and carbohydrates, reduction in the glucose, total cholesterol, and triglyceride content in the blood, etc. [35,36,37,38,39,40,41,42,43,44,45,46,47,48]. The weight-reducing ability of *T. terrestris* plant extracts has been reported [43]. Although the effect of *D. deltoidea* plant extracts on weight during obesity has never been explored, Jeong et al. [90] reported the antiobesity effect of the extract from the closely related species, *D. oppositifolia,* on diet-induced obese mice. The authors observed a decrease in total body weight and parametrial adipose tissue weight, as well as a decrease in total cholesterol, triglyceride level, and LDL-cholesterol in the blood serum. An earlier study of the methanolic extract of *D. nipponica* powder demonstrated its effectiveness against body and adipose tissue weight gains in rodents induced by a high-fat diet [47]. Lee et al. [91] showed that the intake of fresh leaf extracts (3.3 mg/kg/day) or dried leaf extracts (3.3 mg/kg/day) of *P. japonicus* caused a slight decrease in body weight in obese rats, but the effect was statistically insignificant. In the present study, no significant changes in body weight were observed in animals with induced alimentary obesity who received aqueous extracts of biomass of the cell cultures compared to the other groups. However, the intake of *D. deltoidea* cell biomass infusion led to a significant decrease in the body fat mass (up to 27%) of the obese animals and a significant increase in the fat-free mass (up to 11%).

All three preparations of cell biomass showed hypocholesterolemic and hypoglycemic effects that were most pronounced in the animal groups receiving *D. deltoidea* and *T. terrestris* extracts. These data are in agreement with the literature data. For example, *T. terrestris* plant extracts were shown to significantly reduce the level of the total cholesterol and glucose in the blood [41,44]. Similar effects were observed for the extracts of various species of *Dioscorea* also containing steroid glycosides. Several animal studies have shown the antilipemic effects of sapogenin and diosgenin-rich extracts of *Dioscorea* species on hypercholesterolemic animals such as mice and rats, thus resulting in the reduction in the concentrations of blood cholesterol [92]. Another study demonstrated constant improvement in the cholesterol profile of the liver and plasma of mice fed with 50% raw lyophilized yam for 21 days [93]. A dose-dependent glucose-lowering effect on glucose during type 2 diabetes was observed for *P. japonicus* [94].

### 4.2. Effect of D. deltoidea Cell Biomass Preparation on Reproductive Functions of Laboratory Rats

Our study demonstrated that the aqueous extract of *D. deltoidea* cell culture was the most promising for the complex treatment of obesity. Therefore, an additional series of experiments was performed to assess the possible toxic effect of this biotechnologically produced substance on the reproductive functions and offspring development in laboratory rats. Administration of *D. deltoidea* cell biomass at the dose of 100 mg/kg to rats during the period of mating and nursing had no toxic effect on their reproductive functions: the fertility of rats remained 100% and no mortality was observed. At the same time, long-term intake of *D. deltoidea* cell biomass can affect liver function, while de Ritis coefficient values indicate the absence of hepatocellular damage to the liver [95]. Such effects require further research.

After the completion of the lactation period, the health status of both the F0 females and pups was investigated by means of clinical, hematological, biochemical, and pathological anatomical studies. A minor positive effect of *D. deltoidea* cell preparation on blood immune activity was observed as reflected by an increase in the leukocyte and lymphocyte content and hemoglobin content in erythrocytes. A slight shift in the antioxidant system activity in the blood of the F0 animals was also observed, which to some extent was compensated by the proportional increase in glutathione (up to 30%) and catalase (up to 40%) [96,97].

Comparative analysis of the postnatal development data of the F1 offspring from the animals of the experimental parental group consuming *D. deltoidea* phytopreparation revealed a high survival rate, even number of cubs in a litter, and more even distribution of females and males than in the control group, as well as a more intensive weight gain (body weight gain by day 30 was 49% against 36% in the control). The experimental animals showed an increase in total protein and albumin contents (by 7% and 5%) and a statistically significant creatinine increase up to 11%. As for the blood antioxidant potential, there was an increase in total antioxidant activity of 15% and reduced glutathione up to 40%. Taken together, these data may indirectly indicate a more active protein assimilation in the animals of the experimental groups.

## 5. Conclusions

This study presents the results of the preliminary screening of the antiobesity activity of the phytopreparations based on the biotechnologically produced cell cultures of *D. deltoidea*, *T. terrestris,* and *P. japonicus.* Cell culture in vitro is not a full equivalent of the whole plant and, as such, requires thorough investigation and clinical trials before it could be recommended for human treatment.

A comparative evaluation of cell culture extracts in relation to the reference drug liraglutide in the model of alimentary obesity indicated that phytopreparations were efficient in reducing the body fat mass and restored the intracellular-to-extracellular fluid balance in the obese animals. The positive effects were decreasing in the line *D. deltoidea* > liraglutide > intact group > *T. terrestris* > *P. japonicus* > control group (no treatment).

In addition, the aqueous extracts of the cell cultures showed hypocholesterolemic activity which could be ranged by efficiency in the line *P. japonicus* > *D. deltoidea* > *T. terrestris*/liraglutide > intact group > control group. The hypoglycemic effect was most prominent for the cell biomass of *D. deltoidea* and *T. terrestris* and decreased in the order *T. terrestris* > *D. deltoidea* > *P. japonicus* > liraglutide > intact group > control group. Cell preparations influenced the daily diuresis of the animals which increased with the treatment with *P. japonicus* > *T. terrestris* > *D. deltoidea* > liraglutide > intact group > control group. Phytopreparation of *P. japonicus* significantly increased the daily diuresis compared to the control level.

Based on the results presented, the water extract of the *D. deltoidea* cell biomass was the most efficient for the obesity treatment and its positive effects sometimes exceeded those of the reference drug liraglutide.

An additional safety assessment of *D. deltoidea* cell phytopreparation showed no toxic effect on the reproductive function of the animals and their offspring. Therefore, this substance has great potential for the treatment of obesity in women of fertile age.

The experiments presented here should be expanded to test the functional role of biotechnologically derived phytoproducts in other models of obesity to further investigate their effects on induced metabolic disorders. We hope that the results of this study will stimulate a wider application of plant cell cultures as safe and effective supportive remedies for the treatment of obesity and obesity-related complications, particularly during the long-term treatment.

## Figures and Tables

**Table 1:** Animal groups and treatments used in the study.

Group	Subgroup	Feed + Treatment	
		Months 1–3	Months 4–6
Group №1 (intact)	Subgroup №1 (*n* = 10) (intact)	SF	SF
Group №2 (obese)	Subgroup №2 (*n* = 10) (control)	HF	HF, no treatment
	Subgroup №3 (*n* = 10) (reference drug)	HF	HF + liraglutide (0.3 mg/kg)
	Subgroup №4 (*n* = 10)	HF	HF + phytopreparation of *D. deltoidea* (100 mg/kg)
	Subgroup №5 (*n* = 10)	HF	HF + phytopreparation of *T. terrestris* (100 mg/kg)
	Subgroup №6 (*n* = 10)	HF	HF + phytopreparation of *P. japonicus* (100 mg/kg)

SF—standard feed; HF—hypercaloric feed.

**Table 2:** Body measurements of the animals receiving standard or hypercaloric feed for 3 months.

Group	Body Weight (g)	Total Body Water (TBW), %	Extracellular Fluid (ECF), %	Intracellular Fluid (ICF), %	Fat-Free mass (FFM), %	Fat Mass (FM), %	Body Mass Index (BMI)
Standard feed (SF)	275.9 ± 15.13	61.32 ± 2.45	46.10 ± 0.83	53.90 ± 0.81	84.02 ± 3.25	15.98 ± 3.23	8.63 ± 0.34
Hypercaloric feed (HF)	330.9 ± 50.94	49.58 ± 1.28	49.04 ± 0.63	50.96 ± 0.71	64.68 ± 4.02 *	35.32 ± 1.75 *	10.08 ± 0.27 *
*p* Value	*p* = 0.0159	*p* = 0.0004 *	*p* = 0.0283	*p* = 0.0283	*p* = 0.0003	*p* = 0.0003	*p* = 0.0039

*—significantly different at *p* < 0.01.

**Table 3:** Changes in body weight in animals after 6 months of receiving hypercaloric feed with or without liraglutide treatment.

**Group** ^**1**^	**Body Weight (g)**		**Parameter Change**
	Initial	After 6 Months	
Subgroup №1 (intact, SF)	193.11 ± 15.06	373.00 ± 11.24 * *p* = 0.000010	+180.11
Subgroup№2 (obese, HF)	178.67 ± 10.05	419.89 ± 8.86 * *p* = 0.000000 # *p* < 0.05	+241.22
Subgroup №3 (obese, HF + 3 months of liraglutide treatment)	174.30 ± 14.53	289.40 ± 9.96 * *p* = 0.000079 # *p* < 0.05 & *p* < 0.05	+115.10

* Dependent samples t-test # Mann–Whitney U test (against Subgroup №1) & Mann–Whitney U test (against Subgroup №2). ^1^ Group description according to Table 1 SF—standard feed; HF—hypercaloric feed.

**Table 4:** Changes in plasma glucose level in animals after 6 months of receiving hypercaloric feed with or without liraglutide treatment.

**Group** ^**1**^	**Glucose Level in Blood Plasma, mmol/L**		**Parameter Change (Compared to Initial Level)**
	Initial	after 6 Months	
Subgroup №1 (intact, SF)	5.62 ± 0.16	6.13 ± 0.24	+0.51
Subgroup№2 (obese, HF)	5.57 ± 0.25	6.88 ± 0.41 * *p* = 0.036559	+1.31
Subgroup №3 (obese, HF + 3 months of liraglutide treatment)	5.27 ± 0.41	3.97 ± 0.12 # *p* < 0.05 & *p* < 0.05	−1.30

* Dependent samples t-test # Mann–Whitney U test (against Subgroup №1) & Mann–Whitney U test (against Subgroup №2). ^1^ Group description according to Table 1 SF—standard feed; HF—hypercaloric feed.

**Table 5:** Rat fertility (F0) and offspring development assessment (F1) in control group and experimental group receiving *Dioscorea deltoidea* cell biomass for 80 days.

Parameters	Group	
	Experimental (+ *D. deltoidea*)	Control
Reproduction		
Total number of newborn pups	58	66
Litter size at birth, P_25–75_ min–max	8–9 7–9	7–9 4–11
Males/females ratio in the litter, %	53/47	37/63
Pup survival, 1–5 days after birth, %	100	95
Pup survival, 6–25 days after birth, %	100	100
Postnatal development of reflexes, days *		
Surface righting	2–6; 2–4	2–5; 2–3
Negative geotaxis	5–6; 5–5	5–7; 5–5
Auditory startle	8–12; 8–9	8–12; 9–10
Olfactory response	10–12; 10–11	10–12; 10–11
Pupillary function	14–18; 14–15	14–18; 15–16
Visual placing (cliff avoidance)	15–18; 17–18	15–18; 18–18
Bar holding	15–20; 15–17	15–22; 16–18
Accelerated righting	17–22; 17–18	17–22; 17–20

* Data presented as “minimum–maximum; P_25–75_”, where P_25–75_ is the value range from the 25th to 75th percentiles.

**Table 6:** Antioxidant parameters of the blood in the rats of the control and experimental F0 and F1 groups. Parent rats (F0) of the experimental group received the phytopreparation of *Dioscorea deltoidea* cell biomass.

Parameters	Group			
	Experimental F0	Control F0	Experimental F1	Control F1
Total antioxidant activity, Ki 1/(1000 min mL)	**1** **.** **175 ± 0** **.** 012 *	1.261 ± 0.024	**1** **.** **199 ± 0** **.** 201 *	1.022 ± 0.226
MDA, µmol/g of protein	0.832 ± 0.76	0.707 ± 0.065	0.673 ± 0.390	0.692 ± 0.065
Reduced glutathione, mmol/g of protein	**0** **.** **216 ±** **0.** 019 *	0.151 ± 0.011	**0** **.** **114 ± 0** **.** 046 *	0.067 ± 0.038
Catalase activity, U/g of protein	**1** **.** **754 ± 0** **.** 186 *	1.072 ± 0.258	3.247 ± 0.064	3.115 ± 0.058
Glutathione reductase activity, U/L	1410.7 ± 5.5	1389.6 ± 8.6	1097.1 ± 29.4	1140.4 ± 27.2

* significantly different from the control animals at *p* < 0.05. MDA—malondialdehyde content.

## Data Availability

No new datasets were generated during this study. Data sharing is not applicable to this article.

## Appendix A

**Table A1:** White cell distribution in blood of control and experimental groups of F0 and F1 rats. Parent rats (F0) of the experimental group received the phytopreparation of *Dioscorea deltoidea* cell biomass.

Parameters	Reference Values ^1^	Group			
		Experimental F0	Control F0	Experimental F1	Control F1
**White blood cells, 10^9^/** **L**	0.96–7.88	**5** **.** **75 ± 0** **.** **74 ***	4.12 ± 0.69	5.01 ± 0.84	4.15 ± 1.04
Lymphocytes, 10^9^/L	0.68–6.80	4.87 ± 0.66	3.35 ± 0.60	4.25 ± 1.58	3.89 ± 1.36
Monocytes, 10^9^/L	0.02–0.29	0.06 ± 0.02	0.04 ± 0.01	0.16 ± 0.10	0.14 ± 0.07
Granulocytes, 10^9^/L	0.15–1.11	0.76 ± 0.10	0.74 ± 0.04	0.94 ± 0.47	0.56 ± 0.19
Lymphocytes, %	48.9–88.1	81.39 ± 1.27	80.46 ± 1.43	83.50 ± 5.20	82.90 ± 5.10
Monocytes, %	1.3–9.0	1.02 ± 0.19	0.85 ± 0.07	1.23 ± 0.38	0.86 ± 0.16
Granulocytes,%	8.8–43.8	17.40 ± 1.42	18.57 ± 1.48	15.21 ± 5.31	16.24 ± 5.23

* significantly different from the control group at *p* < 0.05 ^1^ according to [98].

**Table A2:** Hematological test panel of control and experimental groups of F0 and F1 rats. Parent rats (F0) of the experimental group received the phytopreparation of *Dioscorea deltoidea* cell biomass.

Parameters	Reference Values ^1^	Group			
		Experimental F0	Control F0	Experimental F1	Control F1
Red Blood Cells Parameters					
Red blood cells, 10^12^/L	7.16–9.24	7.81 ± 0.09	8.02 ± 0.10	8.87 ± 0.60	8.51 ± 0.24
Hemoglobin, g/L	137–172	145.30 ± 1.30	148.00 ± 2.00	153.28 ± 7.14	154.07 ± 6.43
Hematocrit, %	38.5–59.2	**39.96 ± 0.40 ***	41.40 ± 0.52	45.19 ± 3.94	45.02 ± 1.49
Mean erythrocyte volume, µm^3^	50.3–57.0	51.08 ± 0.34	51.71 ± 0.41	51.42 ± 2.17	52.86 ± 1.41
Mean hemoglobin concentration in erythrocyte, g/L	332–378	**363.8** **0** **± 2.7** **0 ***	357.20 ± 2.10	338.54 ± 5.51	340.21 ± 5.47
Red blood cells distribution, %	10.6–14.6	14.91 ± 0.26	14.71 ± 0.12	16.06 ± 0.72	15.81 ± 0.95
Platelets parameters					
Platelets, 10^9^/L	599–1144	631.9 ± 25.2	663.0 ± 21.60	664.38 ± 67.51	627.36 ± 47.94
Plateletcrit, %	-	0.41 ± 0.02	0.43 ± 0.02	0.42 ± 0.05	0.40 ± 0.04
Mean platelet volume, µm^3^	6.4–9.5	6.51 ± 0.04	6.49 ± 0.03	6.39 ± 0.26	6.44 ± 0.2
Platelet distribution, %	-	32.94 ± 0.26	32.69 ± 0.18	32.38 ± 0.9	32.31 ± 0.75

* significantly different from the control group at *p* < 0.05 ^1^ according to [98].

**Table A3:** Biochemical parameters of blood of control and experimental groups of F0 and F1 rats. Parent rats (F0) of the experimental group received the phytopreparation of *Dioscorea deltoidea* cell biomass.

Parameters	Reference Values ^1^	Group			
		Experimental F0	Control F0	Experimental F1	Control F1
Total protein, g/L	57–83	70.63 ± 1.71	69.13 ± 1.15	74.88 ± 5.24	69.41 ± 2.46
Albumin, g/L	37-–58	47.97 ± 1.98	47.99 ± 1.04	50.46 ± 5.27	48.31 ± 3.4
Creatinine, µmol/L	9.0–70.0	56.17 ± 3.10	58.57 ± 1.19	**62.15 ± 3.04 ***	55.43 ± 3.55
Urea, mmol/L	4.28–8.57	**6.95 ± 0.13 ***	6.25 ± 0.20	8.82 ± 0.49	8.19 ± 0.52
Total bilirubin, µmol/L	1.2–3.59	3.10 ± 0.03	2.93 ± 0.16	2.84 ± 0.57	3.03 ± 0.43
Direct bilirubin, µmol/l	0.51–1.20	2.30 ± 0.10	2.27 ± 0.08	2.15 ± 0.36	2.17 ± 0.26
Aspartate aminotransferase, U/L	64–222	110.3 ± 6.4	100.1 ± 5.8	106.22 ± 21.61	128.06 ± 19.19
Alanine aminotransferase, U/L	14–64	**30.59 ± 0.12 ***	25.83 ± 0.17	55.56 ± 3.44	59.14 ± 7.81
Alkaline phosphatase, U/L	62–230	128.0 ± 5.3	118.7 ± 8.6	176.41 ± 30.12	190.26 ± 29.78
Gamma glutamyltransferase, U/L	0–4	2.57 ± 0.29 *	1.66 ± 0.26	1.77 ± 0.59	1.82 ± 0.67
Lactate dehydrogenase, U/L	50–700	281.9 ± 25.2	245.6 ± 17.6	358.03 ± 47.9	341.9 ± 52.42
Total cholesterol, mmol/L	0.60–2.52	2.10 ± 0.06	2.04 ± 0.05	2.31 ± 0.37	2.37 ± 0.40
Triglycerides, mmol/L	0.18–1.93	1.40 ± 0.15	1.11 ± 0.07	1.88 ± 0.45	2.06 ± 0.39
Glucose, mmol/L	3.92–12.21	11.82 ± 0.14	12.39 ± 0.52	10.43 ± 4.1	10.76 ± 3.38

* significantly different from the control group at *p* < 0.05 ^1^ according to [99].

**Table A4:** The relative organ weight (% body weight) in rats of control and experimental F0 and F1 groups. Parent rats (F0) of the experimental group received the phytopreparation of *Dioscorea deltoidea* cell biomass.

Organ	Group			
	Experimental F0	Control F0	Experimental F1	Control F1
Spleen	0.22 ± 0.03	0.23 ± 0.02	0.21 ± 0.02	0.20 ± 0.01
Kidney	0.31 ± 0.03	0.30 ± 0.02	0.28 ± 0.02	0.27 ± 0.01
Liver	3.34 ± 0.18	3.21 ± 0.26	3.12 ± 0.22	2.93 ± 0.16
Heart	0.36 ± 0.04	0.37 ± 0.03	0.29 ± 0.03	0.32 ± 0.03
Thymus	0.13 ± 0.02	0.14 ± 0.03	0.17 ± 0.03	0.19 ± 0.02
